# Adaptive phenotypic plasticity in the Midas cichlid fish pharyngeal jaw and its relevance in adaptive radiation

**DOI:** 10.1186/1471-2148-11-116

**Published:** 2011-04-30

**Authors:** Moritz Muschick, Marta Barluenga, Walter Salzburger, Axel Meyer

**Affiliations:** 1 Lehrstuhl für Zoologie und Evolutionsbiologie, Department of Biology, University of Konstanz, Universitätsstrasse 10, 78457 Konstanz, Germany; 2 Zoological Institute, University of Basel, Vesalgasse 1, 4051 Basel, Switzerland; 3 Dept. Biodiversity and Evolutionary Biology, Museo Nacional de Ciencias Naturales CSIC, José Gutiérrez Abascal 2, 28006 Madrid, Spain

## Abstract

**Background:**

Phenotypic evolution and its role in the diversification of organisms is a central topic in evolutionary biology. A neglected factor during the modern evolutionary synthesis, adaptive phenotypic plasticity, more recently attracted the attention of many evolutionary biologists and is now recognized as an important ingredient in both population persistence and diversification. The traits and directions in which an ancestral source population displays phenotypic plasticity might partly determine the trajectories in morphospace, which are accessible for an adaptive radiation, starting from the colonization of a novel environment. In the case of repeated colonizations of similar environments from the same source population this "flexible stem" hypothesis predicts similar phenotypes to arise in repeated subsequent radiations. The Midas Cichlid (*Amphilophus *spp.) in Nicaragua has radiated in parallel in several crater-lakes seeded by populations originating from the Nicaraguan Great Lakes. Here, we tested phenotypic plasticity in the pharyngeal jaw of Midas Cichlids. The pharyngeal jaw apparatus of cichlids, a second set of jaws functionally decoupled from the oral ones, is known to mediate ecological specialization and often differs strongly between sister-species.

**Results:**

We performed a common garden experiment raising three groups of Midas cichlids on food differing in hardness and calcium content. Analyzing the lower pharyngeal jaw-bones we find significant differences between diet groups qualitatively resembling the differences found between specialized species. Observed differences in pharyngeal jaw expression between groups were attributable to the diet's mechanical resistance, whereas surplus calcium in the diet was not found to be of importance.

**Conclusions:**

The pharyngeal jaw apparatus of Midas Cichlids can be expressed plastically if stimulated mechanically during feeding. Since this trait is commonly differentiated - among other traits - between Midas Cichlid species, its plasticity might be an important factor in Midas Cichlid speciation. The prevalence of pharyngeal jaw differentiation across the Cichlidae further suggests that adaptive phenotypic plasticity in this trait could play an important role in cichlid speciation in general. We discuss several possibilities how the adaptive radiation of Midas Cichlids might have been influenced in this respect.

## Background

Adaptive radiations arise through the rapid divergence of an ancestral species into a multitude of morphologically and ecologically differentiated taxa [1]. This process is assumed to be driven by divergent natural selection and ecological speciation where the adaptation to different niches eventually results in the evolution of reproductive isolation [2]. For example, specialization to certain food resources might lead to divergent habitat preferences, which in turn might isolate the populations reproductively [reviewed in [3]]. Specialization in diet is usually accompanied by morphological adaptations facilitating resource exploitation as has been shown in some textbook examples of adaptive radiation, *e.g*. the Darwin finches on the Galapagos Islands [4], the cichlid fishes in East African lakes [5-7], or the cosmopolitan tiger beetles [8].

Often, adaptive radiations are triggered by an altered adaptive landscape providing opportunity to invade previously not encountered ecological niches (*e.g*. after colonization of a new environment) or not accessible niches (*e.g*. after evolution of a 'key innovation') [9,10]. Recent studies showed that these adaptive peak shifts might happen rapidly [reviewed in [11]], and raise the question of how the adaptive morphological change drives the shift from one peak to another on the adaptive surface [12,13]. Mutation in coding and regulatory sequences and selection might not be sufficient to explain the rapidity of ecological adaptation seen in some instances [14]. Adaptation from standing genetic variation is also not likely to apply to all cases of adaptive radiations, particularly those with only a small number of founders [15]. Adaptive phenotypic plasticity might play a key role allowing populations to enter the 'realm of attraction' of a new adaptive peak, in which genetic assimilation occurs through directional selection favoring genotypes that produce even more extreme phenotypes than what would be possible by plastic response of the ancestral genotype alone [16,17]. Baldwin discussed this topic already in 1896 and described it as 'a new factor in evolution' [18,19]. Although its importance meanwhile became evident, phenotypic plasticity and genetic assimilation were dismissed as being unimportant during the modern evolutionary synthesis [20]. There has been a recent resurgence of interest in these phenomena [21-25], but the link to diversification is still little explored and under debate [26-28]. Not many investigations of phenotypic plasticity in model systems for speciation research, such as cichlid fishes, have been attempted (but see [29-33]).

The Neotropical Midas Cichlid species complex (*Amphilophus *spp.), is recognized among evolutionary biologists for its rapid phenotypic diversification and speciation [6,34]. This species complex has its center of its distribution in Nicaragua, and is comprised of an array of very young species that inhabit both the large Nicaraguan lakes, and several volcanic crater-lakes that contain small scale adaptive radiations [35,36]. The large Nicaraguan lakes, characterized by relatively turbid and shallow waters, have repeatedly acted as source populations for the colonization of nearby crater-lakes newly formed in the calderas of extinguished volcanoes. In these lakes the Midas cichlids encountered novel environmental conditions - *i.e*. presence of deeper zones and clearer water - and speciated *in situ *[34,35,37-41]. Crater-lake species have separated along depth and benthic-limnetic axes [34,35], with the open water column apparently being the first novel habitat invaded. Also, the Midas cichlid species have differentiated in their trophic adaptations. Usage of food sources like stonewort, *Aufwuchs*, evasive invertebrate prey, fish or snails differs species-specifically [39]. The Midas cichlids species, as well as other Neotropical and Old World cichlids, often differ in the relative degree of hypertrophy of a second set of jaws in the throat - the pharyngeal jaw - derived from branchial arch components and important for food mastication [reviewed in [42]]. Specialization for feeding on hard-shelled prey like snails, mussels, or crustaceans (durophagy) through this hypertrophy of the pharyngeal jaw apparatus (PJA) has been found to be a common axis of differentiation in crater-lake Midas cichlids as well as in other cichlid groups [5,31,32,34,42-44]. Its frequency and independency of acquisition across the phylogenetic tree suggests an important role of this adaptation in cichlid speciation [[5], [30], reviewed in [42]]

The Midas cichlid species in the crater lakes are often well differentiated in the trophic apparatus and only a few thousand years old [34-37]. The trophic polymorphism in the Midas crater-lake species could be derived from standing genetic variation, since the polymorphism is present in the large lakes, too [31,32,38,41]. However, the probably limited number of colonizing individuals would render a scenario of the evolution of trait divergence subsequent to colonization also plausible. This scenario is arguably more likely for remote crater-lakes with a monophyletic Midas cichlid assemblage, *e.g*. Lake Apoyo (see [34]). A plausible scenario could be that the divergence in the pharyngeal jaw apparatus in the crater lake Midas cichlid species might have been initiated by phenotypic plasticity in the ancestor. Reproductive isolation might then have occurred via habitat isolation through the heterogeneous distribution of snails in Nicaragua's volcanic crater-lakes, where densities appear to be dependent on depth and substrate type [45]. During times of low food availability otherwise opportunistic individuals adapted for durophagy might confine to areas of high snail density and thereby encounter mates non-randomly in respect to their pharyngeal jaw type [31,32,46,47]. If the ancestor of derived species was phenotypically plastic in ecologically relevant traits, this plasticity might have triggered the diversification. The "flexible stem" model, proposed by West-Eberhard [23], predicts that the directions in phenotypic space in which plasticity is expressed influence the trajectories of phenotypic evolution via genetic accommodation, similar to evolution along "genetic lines of least resistance" [48]. Therefore, it also predicts the outcomes of adaptive radiations seeded by the same ancestor and evolving in similar environments to be similar in terms of their phenotype composition.

In several cichlid fish species (family Cichlidae), plasticity in different traits has been demonstrated: Meyer experimentally induced changes in the oral jaw morphology in the Neotropical cichlid *Parachromis managuensis *by feeding different diets [30], a similar procedure was followed by Bouton and coworkers using the African cichlid *Neochromis greenwoodi *[49]. The Lake Victoria cichlid *Haplochromis pyrrhocephalus *was almost driven to extinction by the upsurge of the introduced, predatory Nile perch in the 1980s, but was able to adapt morphologically to the new environmental conditions of high predatory pressure and eutrophication in only two decades [50]. It has been interpreted that the speed and complexity of these morphological changes relied on a joined action of phenotypic plasticity and genetic change. The molluscivorous *Astatoreochromis alluaudi *naturally exhibits molariform pharyngeal jaws (i.e. stout, broad and strong jaw-bones with wide and flat teeth) [51]. However, when raised on soft artificial food under laboratory conditions [52], in natural conditions in lakes not inhabited by snails [51], or in lakes inhabited by snails but also with a molluscivorous competitor present [53], they develop less stout pharyngeal jaws with cuspid teeth (papilliform).

Specializations matter most during ecological "crunch times", when resource availability is low and opportunistic feeding is precluded [42,46]. The ability to exploit resources then at all or more efficiently than other species can, matters for the individual's survival. But specializations come with a trade-off. The specialization of being able to feed on particular diets especially efficiently often comes at the cost of being much less efficient when dealing with alternative diets. Apparently, such a trade-off exists in the Neotropical Midas Cichlid (*Amphilophus cf. citrinellus*) between two different types of pharyngeal jaws, molariform and papilliform. Individuals with papilliform lower pharyngeal jaws are more effective when dealing with soft food items [54]. Individuals with molariform jaws, on the other hand, can crack larger and harder snail shells and do this faster than papilliform individuals [54].

These cases of phenotypic plasticity, the basis of lacustrine cichlid radiations on trophic specialization [44,55,56] and the possible causal linkage of plasticity and diversification [23,30,31,57] call for examination of adaptive phenotypic plasticity in trophic traits in an adaptive radiation of cichlids comprising species differentiated in these traits. The lower pharyngeal jaw (LPJ) might constitute 'an ideal component of cichlid trophic morphology' to be investigated in this respect [43]. Preferably, the case in study should have a known and young history, involve colonization of new habitats and tests for plasticity in the ancestral or similar to the ancestral source population.

Here, we tested in a common garden experiment the developmental plasticity of the lower pharyngeal jaw of *Amphilophus citrinellus *(Günther, 1864) exposed to diets differing in hardness. Earlier work [31] had suggested that the species in this species complex are phenotypically plastic and that the abundance of molariform fish correlates with the abundance of their major prey item, hard-shelled snails.

The experiment was performed on a laboratory stock derived from the crater Lake Masaya, which was bred in captivity for several decades. Although Lake Masaya is a volcanic crater-lake, its *A. citrinellus *population is very close to the populations of the Lake Nicaragua - which is probably the ancestral source population of most crater-lake radiations - in terms of body shape [35] and phylogenetic relationships [36]. Furthermore, it has been suggested that Lake Masaya might have been colonized as recently as 450 years ago [58].

We investigated whether the development of pharyngeal jaws differed between three types of diets: (1) intact snails with shell, (2) peeled snails without shell, and (3) finely ground up whole snails frozen in pellets, from which fish could nibble off the thawed, soft outer layer when those were given into the water. We aimed to verify whether a hard diet could induce changes in the pharyngeal jaw of the fish, and whether the generation of robust pharyngeal jaws with stout teeth (molariform jaws) was determined by higher calcium content in the diet, or by mechanical stimulation of the jaws when crushing hard food items.

Our study finds that diet can induce changes on the trophic apparatus of the Midas cichlids, and that this changes are related to the mechanical stimulation of the jaws.

## Results

### Geometric morphometric analyses

**Figure 1:**
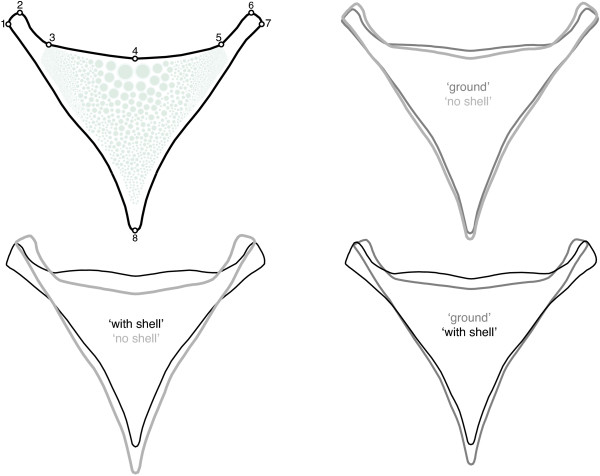
**Induced shape differences**. LPJ shape differences between the diet groups along pairwise discriminant functions depicted as interpolated outlines based on analysis of landmark coordinates. Landmark positions are shown in the upper left. Differences are exaggerated five times for illustration purposes.

**Figure 2:**
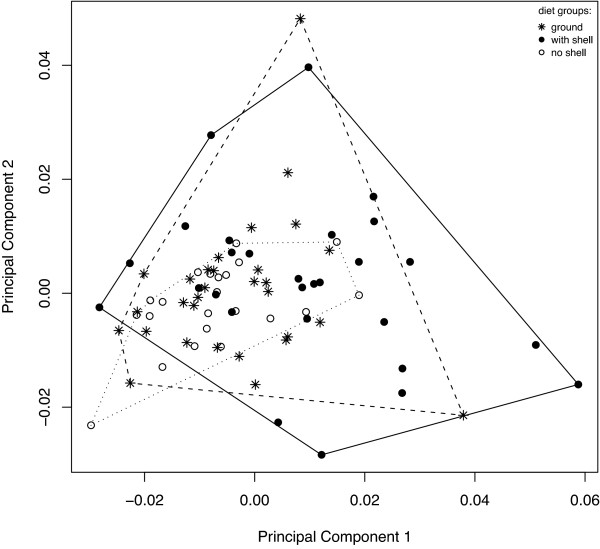
**Morphological separation of treatment groups**. Scatterplot for the first two axes derived from a principal component analysis (PCA) of LPJ landmark data. Percentage of variance explained by the axes is given in parentheses. Note that the large overlap of convex hulls of 'ground' and 'with shell' groups is mainly brought about by two extreme individuals in the 'ground' group.

The shape of the lower pharyngeal jaw differed significantly between the fish raised on a diet 'with shell' and the other two groups of fish as revealed by permutation testing of Procrustes distances (Table 1). The morphological differentiation measured by Procrustes distance was significant and similarly large between the 'with shell' and the two other groups (0.0175 and 0.0135, respectively). The distance between 'ground' and 'no shell' was considerably smaller (0.0067) and not significant. Depicting the between group changes along discriminant functions by warped outline drawings revealed that shape was altered most in functionally relevant regions of the LPJ, namely the posterior horns. In the 'with shell' group the horns (represented by landmarks 1, 2, 6 and 7) pointed more outward and were broader, and jaws were generally shorter along the anterior-posterior axis (Figure 1). Additionally, the posterior outline (represented by landmarks 3, 4 and 5) was less concave in the 'with shell' group as in the other groups. In the 'ground' group the posterior outline was as well less concave as in the 'no shell' group and the horns were directed outward slightly more, but horn width was smaller. The relative overlap on the first two principal components of shape variation between the treatment groups is illustrated in Figure 2.

**Table 1:** Distances in LPJ shape

diet group comparison	procrustes distance	p value
'with shell' vs. 'no shell'	0.0175	<0.0001
'with shell' vs. 'ground'	0.0135	0.0026
'no shell' vs. 'ground'	0.0067	0.15

Distances between the group means in LPJ shape space for data regressed on body weight (Ln). Significance was assessed by permutation testing with 10000 permutations.

### Analyses of weights and lengths

Taking body weight as proxy for ontogenetic stage and correcting for it, measures not covered by the geometric morphometric shape analysis were investigated. The LPJ weight showed significant differences between groups with 'no shell' having the lightest, 'with shell' having the heaviest and 'ground' having intermediate jaws. The centroid size, *i.e*. the scaling factor from the size-removing step in the alignment of landmark configurations, was found to differ significantly between the 'shell' and the 'no shell' group and between the 'shell' and the 'ground' group. Differences were not significant between the 'ground' and the 'no shell' group (Table 2). The dimension not assessed by centroid size, the jaw height, showed no group differentiation if fish body weight was taken as covariate, but showed strong group differentiation when corrected for LPJ weight instead. In that case, the 'no shell' group had the highest, the 'with shell' group the most slender and the 'ground' group intermediate jaws relative to jaw weight. This points to an increase in bone density, moderate with high calcium diet and strong when mechanical impact acted also on the jaws during feeding.

**Table 2:** Group comparisons for morphometric data (non-geometric)

Trait	Factor	p value	WS vs. G	NS vs. G	NS vs. WS
LPJ centroid size	body weight (Ln)	<0.0001			
	diet group	<0.0001	0.036	0.06	<0.0001
	weight × group	0.77			
					
LPJ weight (Ln)	body weight (Ln)	<0.0001			
	diet group	<0.0001	0.018	<0.0001	<0.0001
	weight × group	0.08			
					
LPJ height (Ln)	body weight (Ln)	<0.0001			
	diet group	0.68	0.94	0.67	0.86
	weight × group	0.25			
					
Otolith weight (Ln)	body weight (Ln)	<0.0001			
	diet group	<0.0001	0.40	<0.0001	0.002
	weight × group	0.88			
					
LPJ height (Ln)	LPJ weight (Ln)	<0.0001			
	diet group	<0.0001	0.006	0.0007	<0.0001
	weight × group	0.68			
					
Otolith weight (Ln)	LPJ weight (Ln)	<0.0001			
	diet group	0.0044	0.004	0.71	0.07
	weight × group	0.15			

Results of ANOVAs for length and weight data using diet group and either body weight or LPJ weight as factors. Given are the *p*-values of the ANOVAs and the *p-*values from a subsequent Tukey honest significant difference-test for each group comparison. WS: 'with shell'-group; NS: 'no shell'-group; G: 'ground'-group

**Figure 3:**
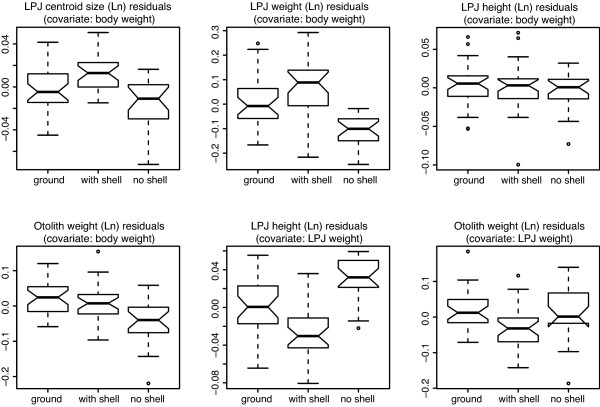
**Character divergence between treatment groups**. Diet group differentiation for regressed morphometric data from LPJs. Regression was either against body weight or LPJ weight. Significance levels are given in Table 2. Boxes range from the lower to the upper quartile and a bar indicates the median. The whiskers exceed the boxes by 1.5 times the inter-quartile-range of the lower or upper quartile, respectively. Notches are a rough proxy for confidence intervals of the median; if they do not overlap between two plots, the medians are most likely significantly different. They extend to +/- 1.58 inter-quartile-range divided by the square root of the number of observations from the median.

The weight of the heavier of the fish's two largest otoliths - the sagittae - using fish body weight as covariate in an analysis of covariance, did not differ in the two high-calcium groups, but was significantly lower in the 'no shell' group (Table 2; Figure 3). Correcting for LPJ weight, the 'with shell' group had significantly lower relative sagitta weight, while 'ground' and 'no shell' did not differ (Table 2; Figure 3).

## Discussion

Phenotypic plasticity has been hypothesized to be able to promote divergence only if it is not complete, *i.e*. sufficient to achieve the same fitness as if the trait was expressed constitutively [20]. A plastic response would be adaptive if it shifts the phenotype in the direction of a new peak on the adaptive surface, and non-adaptive or maladaptive responses to stressful environments would place the phenotype away from any optimum [59]. Here, we were able to induce an adaptive plastic response in the LPJ of *A. citrinellus *by feeding different diets. It qualitatively resembles interspecies differences found in nature, although less pronounced.

In our common garden experiment, the changes induced on the fish exposed to a hard shell diet - *i.e*. horns of the LPJ pointing more outwards, posterior outline less concave, LPJ relatively heavier and possibly increased bone density - mirror those identified as adaptations for mollusk crushing in several other cichlid sister-species pairs [42,43],very closely related species in the Midas cichlid complex in several crater lakes [34,37] and in constitutively expressed [60] or induced [61] phenotypes in other species. The expression of a relatively hypertrophied pharyngeal jaw due to durophagy resembling adaptations found in specialized molluscivorous fish, and the result that hypertrophication is much weaker when fish are fed with high-calcium, low-impact diet leads to the conclusion that the observed phenotypic plasticity is indeed adaptive. The trade-off in feeding performance between different phenotypes further evidences the adaptive nature of plasticity in this trait [54].

A surprising finding is that LPJ height did not differ between the experimental groups, since along this dimension divergence is commonly found in non-molluscivorous/molluscivorous species pairs [53]. A possible explanation would be that this trait behaves allometrically with larger and older molariform fishes expressing more re-growing molars thickening the LPJ. A longer common garden experiment might reveal plasticity in this trait as well. An alternative is that LPJ height is simply not plastic, and its evolution is solely governed by mutation and selection that might bring about developmental constraints. Structural constraints and the lack of phenotypic accommodation would be a possible explanation as well. Under this scenario, an increase in LPJ height would not be possible due to prohibitive spatial demands.

Several findings suggest that no specific and adaptive shape difference was induced by a high-calcium diet alone. Only small differences in shape were observed between 'no shell' and 'ground' groups, and those differences did not resemble known adaptations for durophagy. Furthermore, the comparisons including otolith weight show that calcium allocation is strongly biased towards the LPJ in the 'with shell' group but not in the 'ground' group. There, it appears to affect the skeleton evenly as indicated by the group comparison for sagittae weight when correcting for LPJ weight. This corroborates the finding that the mechanical impact on the LPJ during feeding triggers increased calcium allocation towards the jaw and suggests that a high-calcium diet leads to an unspecific increase in calcium deposition.

The sagittae, as well as the other otoliths, grow in small increments throughout the fish's life [62] and their weight is considered to reflect weight of the individual and availability of calcium during its life. However, Ichii and Mugiya [63] showed that fish raised on a calcium depleted diet did not show different bone densities after a period of 58 days, but were able to substitute the lacking dietary input of calcium by increasing uptake through the gills from the water. Farrell and Campana [64] observed that environmental availability of calcium does not affect its deposition on the otolith. These studies have background levels of calcium in both, supplied diet and water, which might differ from levels in our experiment, involve different species and their experiments were conducted significantly shorter. These differences in experimental setup might explain why in our study an effect of calcium availability on bone and otolith growth was observed as opposed to the other studies.

The effects of the mechanical impact were strong enough to exceed anticipated effects of a higher availability of calcium in the 'ground' diet due to facilitated uptake of minerals from the readily processed shells. 'With shell' fish regularly spat out shell fragments during mastication, and Hoogerhoud [53] reports snail shell pieces to pass the digestive tract of cichlids apparently unharmed. Such observations might explain the slight and non-significant shift towards relatively heavier otoliths in 'ground' fish when accounted for body weight. Several studies on phenotypic plasticity express concerns about the influence of diet quality on developmental differences between treatment groups, so that detrimental effects of a low-quality diet might be mistaken for (adaptive) phenotypic plasticity [33,47,65,66]. Here, we addressed these concerns with our feeding regime. Specifically, we are able to show that induced differences were not due to a lack of calcium in the diet. Even though the studied individuals descended from an inbred line, which has not been subject to artificial selection favoring plasticity in the pharyngeal jaw apparatus, ability to express this trait plastically persisted. This suggests that the plasticity of the LPJ in *A. citrinellus *might not be a trait under selection itself, but more likely an instance of a hidden reaction norm [20].

Similarly to the Midas Cichlid, other cichlid species show PJA adaptable or adapted to durophagy: in Neotropical cichlids non-molluscivorous and molluscivorous species, having papilliform and molariform LPJs respectively, often represent closely related sister species pairs [43]. The same trajectory of divergence has been found between trophic morphs of the same species, *Herichthys minckleyi*, occurring in the Cuatro Ciénegas basin, Mexico. Along the same axis allometric changes happen during the ontogeny of the Mayan Cichlid *Cichlasoma urophthalmus*, introduced in Southern Florida [67]. The presence of hypertrophied pharyngeal jaws is not restricted to cichlids, or even to freshwater fishes: members of the marine families Sciaenidae, Haemulidae and Carangidae express a similar type of PJA, allowing them to feed on hard-shelled prey. The phylogenetic relationship to species with non-hypertrophied pharyngeal jaws can be close, *e.g*. congeneric, in these cases as well [68].

The number of cases of closely related species or trophic morphs of a single species exhibiting such divergent morphologies, as well as their phylogenetic dispersal, is astonishing. This trajectory in morphospace might be similarly important as the well-known deep-bodied vs. elongated body trajectory found in many benthic-limnetic fish species pairs (*e.g*. [69-71], and those reviewed in [72]). Both phenotypic contrasts are usually accompanied by extensive diet and/or habitat preference differences, respectively. Such ecological diversification has been shown to be a major factor in empirically studied speciation events and its importance in speciation is well supported by theoretical models [34,73-75]. In the Midas Cichlid species complex, ecological diversification has been shown to occur along both axes, even in correlation [31], and probably led to speciation in several cases [34].

### Phenotypic plasticity and rates of diversification

The importance of phenotypic plasticity in population divergence and speciation gained increasing attention in the last years [22,23,26,33,47,57,76-81]. Both studies focusing on single species and studies within a larger comparative framework investigated this link: Nylin & Wahlberg found support for a 'plasticity scenario' for the diversification of nymphaline butterflies during the Tertiary and argued that herbivorous taxa able to occupy several niches were more likely to diversify along with the angiosperm radiation [82]. In coastal San Diego a population of montane dark-eyed juncos (*Junco hyemalis*, Aves) was able to establish itself due to an adaptive plastic response in reproductive effort [83]. A recent review by Pfennig *et al*. [57] summarizes theoretical and empirical studies and diagnoses an important, but largely underappreciated, role of phenotypic plasticity in speciation and adaptive radiation. Comparing sister clade pairs - with one clade being known to include cases of resource polyphenism, while the other does not - Pfennig and McGee found evidence that resource polyphenism is associated with greater species richness in fishes and amphibians [28].

The role of phenotypic plasticity in population divergence appears to be at least twofold: (1) plasticity increases the probability of population persistence after colonization of a new environment, thus making its split from the ancestral population more likely [83,84], and (2) plasticity provides means of conquering other peaks on the adaptive landscape, possibly leading to assortative mating and speciation with parallel outcomes in repeated cases [12,14,17,23,33].

Theoretical investigations support these predictions. Probability of population persistence increases with plasticity while being dependent on the amount of environmental change and the costliness of plasticity [85]. At a moderate rate of environmental change and if plasticity is costly, high levels of plasticity are expected to lead to an increased probability of extinction while an intermediate level improves the ability of persistence [85]. Access to novel ecological niches is improved because an increase in epigenetic variability does facilitate the circumvention of adaptive valleys and smoothes the fitness landscape [13,86,87]. Using numerical simulations Thibert-Plante and Hendry [26] find plasticity to commence reduction in gene flow between populations in contrasting environments. To do so, plasticity must occur before dispersal but could then lead to reproductive isolation even prior to any adaptive genetic divergence.

Our demonstration of adaptive phenotypic plasticity in the LPJ of *A. cf. citrinellus *suggests that this could be a crucial factor in ecological speciation and adaptive radiation in the repeated *Amphilophus *crater-lake radiations and possibly in other cichlid clades as well [30,31]. The results of our experiment support the "flexible stem" hypothesis, in that the induced differences between treatment groups - more robust LPJs in the 'with-shell' group, less robust LPJs in groups fed soft food - resemble between-species differences in crater-lake radiations. However, we did not test for plasticity in the ancestor itself, nor in fish derived from the large Nicaraguan Lakes, but in a stock derived from Lake Masaya. The different history might have caused an alteration of the plastic response in experimental groups compared to the real ancestor. But since there is a considerable chance of the Lake Masaya *A. citrinellus *population being very young and since plasticity here seems not be lost easily (at least not over several generations), we suggest that our results endorse the "flexible stem" hypothesis for the Midas Cichlid assemblage. Because the induced plasticity does not reach the extent of morphological divergence found between species in nature we conclude, that the expectations from the "adaptive surface model" are fulfilled as well.

In which way exactly phenotypic plasticity and genetic accommodation in the pharyngeal jaw might abet diversification in the *Amphilophus *species complex remains speculative. A direct influence on the formation of reproductive isolation might be given through enhancement of habitat preference. If individuals expressing the same type of pharyngeal jaw have a higher chance of mating with each other, and gene flow between groups is hampered strongly enough, population subdivision might be initiated. The heterogeneous distribution of snails, if it is stable over time and patches are sufficiently large, might be the basis for habitat preference by jaw type. Alternatively, the hypothesized function of the pharyngeal jaw apparatus in sound production, *e.g*. during courtship, might bring about assortative mating according to jaw type if female sound preference is divergent as well [88].

However, even if phenotypic plasticity is less important in sympatric speciation scenarios it might still influence diversification in allopatry [reviewed in [89]]. By augmenting the probability of population persistence after colonization of a new environment, *e.g*. a crater-lake, and the possibility of genetic accommodation of plastic trait changes the likelihood of allopatric speciation between ancestral source population and the new colonizing population is increased. It remains unclear, whether or not the repeated endemic radiations of Midas cichlids in Nicaraguan crater-lakes are facilitated by phenotypic plasticity in the pharyngeal jaw or if the constitutively expressed differences in jaw shape between species are a secondary result of speciation driven by other factors. The best documented case of an in-crater-lake diversification, the origination of the Arrow cichlid *Amphilophus zaliosus *in Lake Apoyo, seems to have been driven by diverging habitat preferences with differences in pharyngeal jaw shape being probably secondary [34]. However, in other, less-well documented cases the hypothesis that adaptations in the pharyngeal jaw apparatus triggered divergence remains valid, but would need to be further investigated.

## Conclusions

We demonstrated phenotypic plasticity in the pharyngeal jaw of the cichlid fish *Amphilophus citrinellus *that is due not to differences in nutritional composition of the diet, but brought about largely by the mode of feeding. This finding might suggest that plasticity plays an important role in diversification.

Future research on how a plastic reaction in one trait could impact the expression of other traits through correlated plastic responses might contribute to the understanding of parallelisms so often encountered in nature. For example, it seems the papilliform pharyngeal jaw type is correlated with fusiform limnetic body shape whereas the molariform jaw type is correlated with deeper, benthic body shape [31]. The extent to which this 'integration of plastic responses' [81] is determined, and by which factors, still remains to be elucidated. Also, what role a stage of fixed polymorphism plays in the process of diversification, whether it is an intermediate step [42] or a 'dead-end', remains to be investigated.

How adaptive phenotypic plasticity is mediated genetically is another important issue. In cichlids, the family of bone morphogenetic proteins (BMPs) is known to be involved in shaping bones of the oral and pharyngeal jaws [90] and might constitute good candidates, along with respective transcription factors and ligands, for the elucidation of the genetics of phenotypic plasticity in the PJA.

Cichlids, are a prime system for speciation research and have an important trophic trait expressed plastically, and therefore constitute a cogent group for investigating the role of adaptive phenotypic plasticity in diversification. Research combining experimental and field studies with modern tools of analysis, such as sensitive group assignment methods or gene expression quantification, will be most rewarding avenues of research to elucidate the link between plasticity and speciation

## Methods

### Common garden experiment

We divided fry of a single *Amphilophus citrinellus *brood from an inbred line into three similarly sized groups and fed them on diets differing in mechanical durability and calcium content. The three study groups of 30 *A. citrinellus *individuals each were kept under standardized laboratory conditions with 12 h daylight for a period of six month. The fish stock used (AM-stock at the University of Konstanz) derives from Lake Masaya, a volcanic crater-lake in Nicaragua. Originally, these fish came from the Berkeley stocks of George Barlow who gave some of these fish to the Steinhard Aquarium in San Francisco. In 2001 fish from there were brought to Konstanz and are the stock of *A. cf. citrinellus *that were used in these experiments. This fish stock has been bred in captivity on soft artificial food for several decades. Moreover, in Lake Masaya no snails occur and neither are cichlids with molariform pharyngeal jaws reported [54,91].

The fish groups were raised on different diets: (1) *Melanoides tuberculata *snails, laboratory grown, with intact bodies and intact or slightly damaged shells (in case the snail was deemed too large), (2) snail bodies, where the shells were manually removed, and (3) *M. tuberculata *with shell but ground to fine paste using mortar and pestle, which was given frozen in pieces to large to be swallowed as a whole. Food amount was adjusted to match group's estimated size gain. Fish were kept in one large tank (1.8 × 0.5 × 0.5 meter, 450 l) and perforated walls allowed water exchange between the compartments containing the three experimental groups. To counteract position bias, we swapped groups between compartments several times throughout the experiment.

### Measurements & analyses

Fishes were sacrificed and weighed, and standard and total length were recorded. We excised LPJs and sagittae, and cleaned and dried them. LPJs and otoliths were weighed to the nearest milligram. LPJs were scanned on a standard desktop scanner. Coordinates of 8 landmarks were recorded for each LPJ using tpsDig 2.11 ([92], for landmark positions see Figure 1). Landmarks represented homologous, defined locations on the jaws outline. Their positioning followed Klingenberg *et al*. [93] with the exception of their landmarks 5 and 6 - instead the anterior tip was covered by our landmark 8. Otherwise landmark position were the same, though differently numbered. Landmark arrangements were procrustes aligned, *i.e*. their positional, rotational and size information was removed from the dataset. However, size information was recorded in centroid size and was used for joint analysis with other data. Since the LPJ is a symmetrical structure we extracted the symmetric component of shape variation using MorphoJ [94]. We conducted discriminant function analyses (DFA) for each pair of groups to produce Figure 1. A canonical variates analyses (CVA) using residuals of a pooled-within-diet-groups regression on body weight (Ln) yielded mean shape distances and their significance levels were assessed by permutation testing (10.000 permutations).

Fish body weight, LPJ weight, height, and centroid size, and otolith weight were evaluated via analysis of variance (ANOVA) and group-pairwise differences of residuals means were assessed for significance using Tukey's honest significant difference-test. All these measures were Ln transformed prior to analysis. For otoliths the weight of the heavier sagitta was used, to minimize influence of preparation damage.

All statistical tests on length and weight data were performed using the R statistical environment [95].

## Authors' contributions

MM participated in conceiving the study and the experimental design, ran the experiment, gathered the data, analyzed the data and drafted the manuscript. MB participated in conceiving the study and the experimental design and helped with gathering data and preparation of the manuscript. WS participated in conceiving the study and the experimental design and helped with preparation of the manuscript. AM participated in conceiving the study and the experimental design and helped with preparation of the manuscript. All authors read and approved the final manuscript.

## Authors' information

MM was a Master's student in AM's laboratory and is now a Ph.D. student with WS and interested in the phenomenon of convergent evolution and its implications for speciation and adaptive radiations.

MB was a postdoc in AM's laboratory when this study was conducted. She is an evolutionary ecologist interested in speciation and the origin of adaptive radiations.

WS was a postdoc in AM's laboratory when this study was conducted. He is an evolutionary biologist interested in the evolution of adaptive radiations of cichlid fishes.

AM is an evolutionary biologist interested in speciation and the origin of evolutionary diversity.
